# Photon Detection as a Process of Information Gain

**DOI:** 10.3390/e22040392

**Published:** 2020-03-30

**Authors:** J Gerhard Müller

**Affiliations:** Department of Applied Sciences and Mechatronics, Munich University of Applied Sciences, D-80335 Munich, Germany; gerhard.mueller@hm.edu

**Keywords:** photon, photon detection, information gain, detection efficiency, figure of merit (FOM), Landauer principle

## Abstract

Making use of the equivalence between information and entropy, we have shown in a recent paper that particles moving with a kinetic energy ε carry potential information $i_{pot}\left(\varepsilon ,T\right)=\frac{1}{\ln\left(2\right)}\frac{\varepsilon }{k_{B}\;T}$ relative to a heat reservoir of temperature T. In this paper we build on this result and consider in more detail the process of information gain in photon detection. Considering photons of energy $E_{ph}$ and a photo-ionization detector operated at a temperature $T_{D}$, we evaluate the signal-to-noise ratio $SN\left(E_{ph},T_{D}\right)$ for different detector designs and detector operation conditions and show that the information gain realized upon detection, $i_{real}\left(E_{ph},T_{D}\right)$, always remains smaller than the potential information $i_{pot}\left(E_{ph},T_{D}\right)$ carried with the photons themselves, i.e.,: $i_{real}\left(E_{ph},T_{D}\right)=\frac{1}{\ln\left(2\right)}\ln\left(SN\left(E_{ph},T_{D}\right)\right)\leq i_{pot}\left(E_{ph},T_{D}\right)=\frac{1}{\ln\left(2\right)}\frac{E_{ph}}{k_{B}T_{D}\;}$. This result is shown to be generally valid for all kinds of technical photon detectors, which shows that $i_{pot}\left(E_{ph},T_{D}\right)$ can indeed be regarded as an intrinsic information content that is carried with the photons themselves. Overall, our results suggest that photon detectors perform as thermodynamic engines that incompletely convert potential information into realized information with an efficiency that is limited by the second law of thermodynamics and the Landauer energy bounds on information gain and information erasure.

## 1. Introduction

Ever since the discovery of the second law of thermodynamics and the formulation of Maxwell’s demon paradox [1], researchers have been actively searching for connections between entropy and information. After more than a decade of discussions [2,3,4], an equivalence between information and entropy [5,6,7,8,9] appears to be taken for granted. Gaps in our understanding, nevertheless, remain as thermodynamics in its classical form does not mention information at all and information in the eyes of many researchers still continues to look more like a conceptual vehicle rather than a true physical quantity. Parrondo, Horowitz and Sagawa [10] therefore suggest that reconciliation of the information/entropy relationship with classical thermodynamics will require thermodynamic laws to be restated in a way that incorporates information explicitly [11,12,13,14,15,16] and that the physical nature of information ultimately needs to be clarified to ensure that it enters thermodynamic laws as a physical entity rather than in the form of a mathematical abstraction [17,18].

In the present paper we discuss the idea that photon detectors may be regarded as thermodynamic engines that incompletely convert potential information

(1) $$i_{pot}\left(E_{ph},T_{d}\right)=\frac{1}{\ln\left(2\right)}\frac{E_{ph}}{k_{B}T_{D}\;},$$

initially carried with photons of energy $E_{ph}$ themselves, into pieces of realized information, $i_{real}\left(E_{ph},T_{D}\right)$, as these interact with detectors operated at a temperature $T_{D}$. These ideas of potential and realized information have arisen out of a recent paper [19] in which the equivalence between information and entropy had been used to interpret the entropy of a molecular gas as information that is carried with the individual molecules but which remains missing and inaccessible to external observers. In the present paper, we want to test these ideas by studying the process of information gain in a practically important and well-understood class of sensing devices.

The class of sensing devices we want to consider are photon detectors which play important roles in diverse fields of application. To date, photon detection is considered to be a well-developed and mature technology, supported by a firm theoretical basis which is summarized in excellent textbooks [20,21,22]. The related technical theories accurately account for the performance of photon detectors in terms of detector material properties and detector operating conditions and these fully satisfy all practical engineering demands. As all kinds of photon detectors ultimately provide electrical output signals, all known figures of merit (FOM) for photon detectors such signal-to-noise ratio, noise-equivalent power and detectivity relate to such output signals. Interestingly, and similar to classical thermodynamics, these technical theories do not mention information at all, although photon detectors very obviously are information-generating devices. Furthermore, these theories remain vague with regard to the concept of detection events.

The point we want to make in this paper is that the concepts of information and detection events can easily and explicitly be introduced into the existing theories of the photon detector response in the form of physically measurable quantities. In order to arrive at this goal, we consider in Section 2 and Section 3 the detection of photons with the help of photo-ionization detectors (PID). There, we show that the information gain, $i_{real}\left(E_{ph},T_{D}\right)$, that can be realized upon detection of a photon of energy $E_{ph}$, always remains smaller than the potential information $i_{pot}\left(E_{ph},T_{D}\right)$ that is carried with the photons themselves, i.e.,:

(2) $$i_{real}\left(E_{ph},T_{D}\right)=\frac{1}{\ln\left(2\right)}\ln\left(SN\left(E_{ph},T_{D}\right)\right)\leq i_{pot}\left(E_{ph},T_{D}\right)=\frac{1}{\ln\left(2\right)}\frac{E_{ph}}{k_{B}T_{D}\;}.$$

In performing this analysis, the specific choice of PIDs was motivated by the fact that the limit performance of PIDs matches the performance of ideal photon detectors as defined in the textbook of Kingston [20]. As such ideal detectors generate the maximum conceivable information gain, Equation (2) supports the idea that the information $i_{pot}\left(E_{ph},T_{D}\right)$ does indeed represent an information content that is intrinsically carried with the photons themselves and that can only partially be retrieved when the photons interact with detectors operated at temperatures $T_{D}$, satisfying $k_{B}T_{D}\ll E_{ph}$. The considerations in Section 4 then reveal that potential information relates to the potential of photons of generating entropy, i.e., missing information, as these become absorbed inside macroscopic bodies such as photon detectors. Realized information, on the other hand, is associated with macroscopically observable signal transients which are produced as photon energy is dissipated and broken down into smaller pieces of size $k_{B}T_{D}$. As physical entities, detection events reveal as pieces of physical action that are generated at the expense of energy dissipation and which are endowed with a finite observational value that is measured by the relative magnitude of the realized to potential information, $\eta_{D}=$ $i_{real}/i_{pot}$.

## 2. Photo-Ionization Detection

**Figure 1:**
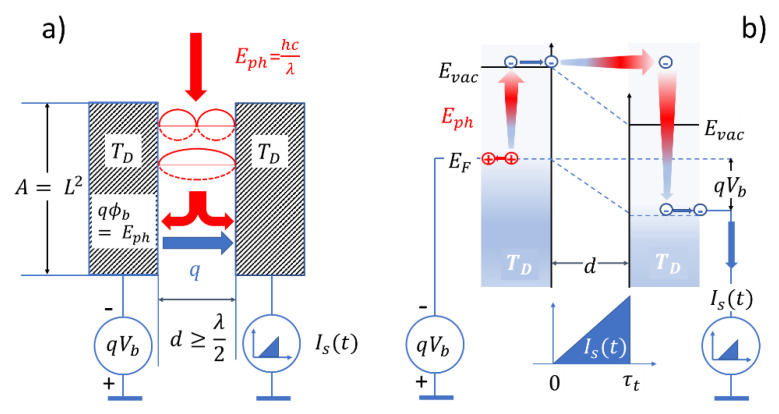
Photo-ionization detector (PID): (**a**) geometrical arrangement; (**b**) energy band model. The red arrows in (**a**) indicate signal photons. The blue arrow indicates a photoelectron excited by photon absorption in the negatively biased emitter electrode and following the electrical field lines towards the grounded collector electrode. The arrows in (**b**) denote electron transit paths induced by photoionization, acceleration through the detector gap and thermalization upon absorption inside the collector electrode.

The device we want to consider in our analysis of the photon detection process is shown in Figure 1a. Two metal plates with area $A=L^{2}$ are positioned face-to-face to each other at a distance d to form a parallel-plate capacitor. From the top side, photons with energy $E_{ph}$ and wavelength $\lambda =hc/E_{ph}$ are allowed to enter the gap between both plates in which the photon wavefields become guided until they exit at the lower end. Assuming that the photon energy exceeds the work function of both metal plates, i.e., $E_{ph}\geq \;q\phi_{m}$, electrons inside the metal plates may get excited from their respective Fermi energies $E_{F}$ to their vacuum levels $E_{vac}$, from where they are free to move into the gap separating both electrodes. In case an electrical bias $V_{b}$ is applied across this gap, a directional electron current $I_{s}\left(t\right)$ is induced that flows from the negatively biased emitter to the grounded collector electrode where the current flow can be monitored. As indicated in the band diagram of Figure 1b, electrons emitted with zero speed from the emitter electrode pick up speed inside the gap thereby producing triangular current pulses which abruptly end when the photoelectrons arrive at the collector surface. Once absorbed there, the electrons thermalize through a huge number of unoccupied electron states until they end up at the Fermi energy in this electrode. In this thermalization process, all kinetic energy that had been gained inside the detector gap is dissipated and a small amount of energy is added to the huge internal energy in this electrode without producing any measurable temperature change. Such electron pulses, produced at the expense of energy dissipation, obviously form the observational images of photon–detector interactions. Once such an event has been observed, however, this does not uniquely proof that a true photon–detector interaction has taken place, i.e., that an externally generated photon had triggered the observed event. This problem of lowered observational value of events arises from the fact that the emitter and collector electrodes are operated at a finite temperature $T_{D}$, which causes them to emit blackbody radiation with photon energies $E_{ph}\geq \;q\phi_{m}$ into the detector gap. As the photoelectron transients, triggered by such internally generated photons, cannot be distinguished from transients that had originated from externally generated ones, the observational value of each observed detection event is compromised and lowered to some extent. Meaningful measurements, obviously, can only be carried out in the case that the number of signal electrons $N_{s}$ in each observational time interval Δt exceeds the number of noise electrons $N_{n}$, i.e., when the signal-to-noise ratio is significantly larger than one: i.e., $SN=N_{s}/N_{n}\gg 1$.

In Section 3 we analyze the processes of signal and noise current generation in a more quantitative manner using the standard theory of PID detectors [20] to arrive at formulae which are needed in Section 4 to arrive at an informational reformulation of the PID response theory and its generalization to other kinds of photon detectors. Considering our intention of testing the validity of Equation (1), i.e., finding evidence for an intrinsic information content that is carried with photons themselves, we concentrate on processes of single-photon detection.

## 3. Analysis of PID Performance

### 3.1. Signal Currents

In the following we assume that both electrode work functions exactly match the energy of the photons to be detected, i.e., $E_{ph}=q\phi_{m}$. In this case, electrons excited to $E_{vac}$ start out on the emitter side with zero initial speed and follow the electrical field lines across the detector gap, picking up speed according to

(3) $$v\left(t,d,V_{b}\right)=\frac{q}{m_{e}}\frac{V_{b}}{d}t,$$

until they finally arrive at the collector electrode with speed $v\left(\tau_{t}\right)$ at time $\tau_{t}$:

(4) $$v\left(\tau_{t},d,V_{b}\right)=\sqrt{\frac{2qV_{b}}{m_{e}}}=2v_{av}$$

(5) $$\tau_{t}\left(d,V_{b}\right)=\frac{d}{v_{av}}=\frac{d}{c}\sqrt{\frac{2m_{e}c^{2}}{qV_{b}}}.$$

In these latter equations $v_{av}$ stands for the average speed of the electron within the gap, $m_{e}$ for the electron rest mass, q for the elementary charge and c for the speed of light.

Considering that signal photons can become absorbed with equal probability in the emitter and collector electrodes and that only photoelectrons excited inside the negatively biased emitter electrode can follow the electrical field lines through the gap, single electrons travelling across the detector gap produce triangular current pulses with time duration $\tau_{t}$ amounting to

(6) $$I_{s}\left(t,\;d,V_{b}\right)=\frac{1}{2}q\frac{v\left(t\right)}{d}=q\frac{t}{\tau_{t}{\left(d,\;V_{b}\right)}^{2}},\;(0\;\leq t\;\leq \;\tau_{t}).$$

As such current transients are the observational images of photon–detector interactions, these constitute detection events. In Section 4.1 we demonstrate that such events also represent pieces of physical action, produced at the expense of energy dissipation.

### 3.2. Noise Currents

**Figure 2:**
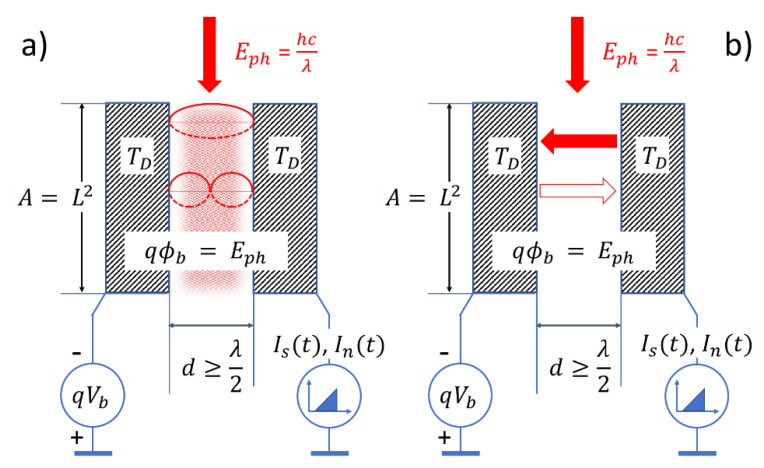
PID illustrating the competition of externally generated signal photons (vertical arrows) with internally generated noise photons: (**a**) detector gap filling with internally generated thermal noise photons (red cloud); (**b**) efficiency of thermal noise photons in generating noise photoelectrons as emitted from the collector (full arrow) and emitter sides (empty arrow).

Figure 2 schematically illustrates the competition of externally generated signal photons and internally generated noise photons. While Figure 2a shows that both electrodes emit blackbody radiation into the detector gap, Figure 2b indicates that only photons emitted from the negatively biased collector towards the grounded emitter electrode can follow the electrical field lines through the detector gap and thereby trigger photoelectrons and observable current transients.

In order to arrive at the signal-to-noise ratio, we consider a situation in which the signal source of photons emits photons of energy $E_{ph}$ at an average rate of one photon per electron transit time $\tau_{t}$ in the detector. In addition to these signal photons, thermally generated noise photons with energies $E_{ph}\geq q\phi_{m}$ are emitted from the collector towards the emitter electrode. Their fraction can be found by integration of the Planck blackbody distribution starting from $E_{ph}$ and extending to infinity [20]:

(7) $$n_{th}\left(E_{ph,}T_{D}\right)=\frac{2\;\pi }{c^{2}h^{3}}{\left(k_{B}T_{D}\right)}^{3}exp\left[-\frac{E_{ph}}{k_{B}T_{D}}\right]\left\{{\frac{E_{ph}}{{\left(k_{B}T_{D}\right)}^{2}}}^{2}+2\frac{E_{ph}}{k_{B}T_{D}}+2\right\}.$$

With the density of photons per unit area and unit time being known, the total number of noise electrons emitted into the detector gap during a single electron transit time becomes

(8) $$N_{th}\left(E_{ph},T_{D},A,\tau_{t}\right)=A\;\tau_{t}\left(d,V_{b}\right)\;n_{th}\left(E_{ph,}T_{D}\right).$$

As emission and absorption processes occur at random times, the number of internally generated noise electrons will suffer statistical fluctuations with a dispersion of

(9) $$\Delta N_{th}\left(E_{ph},T_{D}\right)=\sqrt{N_{th}\left(E_{ph},T_{D}\right)},$$

which yields for the signal-to-noise ratio:

(10) $$SN\left(E_{ph},T_{D},d,V_{b}\right)=\frac{N_{s}}{N_{n}}=\frac{1/2}{\Delta N_{th}}=\frac{1/2}{\sqrt{A\;\tau_{t}\left(d,V_{b}\right)\;n_{th}\left(E_{ph,}T_{D}\right)}}.$$

### 3.3. Physical Limitations to the PID Response

Looking back on Equations (6) and (10), it is seen that all relevant detector performance parameters depend on the magnitude of the electron transit time $\tau_{t}\left(d,\;V_{b}\right)$ through the detector gap. As the magnitude of $\tau_{t}\left(d,\;V_{b}\right)$ can be controlled either through the detector design (d) or by adjustment during detector operation ($V_{b}$), it is relevant to ask for the physical limitations that exist with regard to these two parameters.

Turning to d first, we note that the detector gap principally performs as a waveguide for the photons to be detected (Figure 1a). This implies that the cutoff photon wavelength $\lambda =hc/E_{ph}$ sets a lower physical limit to d [23]. As the confinement of the photon wavefields inside the detector also depends on the size of the emitter and collector electrodes, similar limitations apply for the minimum electrode size $L_{min}$ as well [23]:

(11) $$d_{min}=L_{min}=\frac{\lambda }{2}=\frac{hc}{2E_{ph}}.$$

Reducing the detector gap width below this limit, the gap becomes too small to propagate photon wave fields with wavelengths $\lambda \geq d_{min}$ through the gap. At the limit $d=d_{min}$, signal photons just remain able to penetrate the detector gap and to produce photoelectrons. Similarly, when the lateral dimensions of the emitter and collector electrodes are reduced below $L_{min}$, photon wave fields are no longer effectively confined within the gap, which lowers the ionization probability as well. 

With the detector gap width d and the electrode size L having been fixed at their minimum values, the signal current and its time response can be further increased by increasing the bias voltage $V_{b}$. An upper physical limit of $V_{b}$ is reached when the bias voltage is increased to

(12) $$qV_{b\_max}=2m_{e}c^{2},$$

i.e., to a level at which electrons travelling through the detector gap will impact on the collector electrode with energies high enough to create electron–positron pairs [24,25]. As the positrons are able to travel back through the detector gap towards the emitter electrode, gaining at the same time enough energy to generate follow-on electron–positron pairs upon electrode impact, an exponentially increasing avalanche of charge carriers is initiated, which makes the detector gap electrically conductive without any photon energy input. At such bias levels, the range of useful device operation is obviously exceeded.

With the above values of $d_{min}$ and $V_{b\_max}$, Equations (5), (6) and (10) can be re-written as

(13) $$\tau_{t}\left(d,V_{b}\right)=\tau_{min}S\left(d,V_{b}\right)$$

with

(14) $$\tau_{min}=\frac{1}{2}\tau_{ph}=\frac{1}{2}\frac{h\;}{E_{ph}\;}$$

and $\tau_{ph}$ standing for the photon vibrational period, and

(15) $$S\left(d,V_{b}\right)=\left(\frac{d}{d_{min}}\right)\sqrt{\frac{V_{b\_max}}{V_{b}}}\geq 1.$$

This latter function collects all experimentally controllable parameters and determines how far the electron transit time $\tau_{t}$ is elongated above its minimum value of $\tau_{min}$.

Returning to Equations (11) and (14), it can be observed that operating the detector at its ultimate physical limits of $S\left(d_{min},V_{b\_max}\right)=1$, the photons to be detected are confined in space and time within the detector gap to an extent that is limited by the position–momentum and time–energy uncertainty relationships:

(16) $$\left(\frac{\lambda }{2}\right)\left(\frac{E_{ph}}{c}\right)=\frac{h\;}{2\;},\;\mathrm{and}:\;\;\tau_{t}\;E_{ph}=\frac{h}{2}.$$

With these minimum values of d, L and $\tau_{t}\left(d,V_{b}\right)$ and in the limit $E_{ph}\gg k_{B}T_{d}$ the signal-to-noise ratio becomes:

(17) $$SN\left(E_{ph},T_{D},V_{gap},V_{b}\right)=\frac{1}{\sqrt{\pi }}\sqrt{\frac{\left[\frac{E_{ph}}{k_{B}T_{D}}\right]exp\left[\frac{E_{ph}}{k_{B}T_{D}}\right]}{\left[\frac{V_{gap}}{V_{min}}\right]\sqrt{\frac{V_{b\_max}}{V_{b}}}}}.$$

This latter equation shows that the signal-to-noise ratio is optimized when the detector operation temperature is reduced as much as possible and when the size of the detector gap $V_{gap}=L^{2}d$ is reduced to the smallest possible size, $V_{min}=L_{min}{}^{2}d_{min}={\left(\lambda /2\right)}^{3}$, that still allows photons of energy $E_{ph}=hc/\lambda$ to be confined within this gap [23]. Furthermore, the bias potential applied across the detector gap needs to be increased towards its maximum size to make the photoelectrons move at the same speed as the photon wave fields that had produced them, i.e., $v_{el}=c$. Clearly, such extreme conditions cannot be met in any technologically realizable device [20], but these are the ultimate physical limits that might be approached in principle.

## 4. Informational Reformulation of Detector Response Theory

So far, we have been considering the standard theory of PID detectors as described in the textbook of Kingston [20]. As already stated in the introduction, this theory successfully satisfies all engineering demands concerned with the development and use of such photon detectors. This standard theory, however, completely disregards the concept of “information” and it remains vague concerning the concept of “detection events”. In the following, both conceptual vehicles are discussed and introduced into the theory as physically measurable quantities.

### 4.1. Detection Events and Entropic Cost

As described above, triangular current pulses of the form

(18) $$I_{D}\left(t,\;d,V_{b}\right)=q\frac{t}{\tau_{t}{\left(d,\;V_{b}\right)}^{2}},\;(0\;\leq t\;\leq \;\tau_{t}).$$

form the observational images of photon–detector interactions. As it does not matter whether these pulses had been caused by true photon–detector interactions, i.e., by signal photons, or by internally generated noise photons, we use the more general index D=detection, here. Both kinds of current transients constitute detection events, however, with a limited observational value as by simple observation it does not become clear whether an observed event has been a signal or a noise event.

In addition to time-dependent functions like Equation (18), such current transients can also be characterized in an integral, per-event manner by evaluating integrals over the entire pulse duration. A first and obvious opportunity is integrating the current transient over the time interval $0\;\leq t\;\leq \;\tau_{t}$ to determine the total collected charge. In case this charge happens to coincide with the elementary charge q, evidence is provided that a single photon has been detected.

Further integral quantities that can be derived from such single-electron transits can be obtained by multiplying the current pulses $I_{D}\left(t,\;d,V_{b}\right)$ with the bias potential $V_{b}$ through which the photoelectrons had fallen during their transit through the detector gap. In this way the signal power $P_{D}\left(t,\;d,V_{b}\right)$ is obtained. Double integration over time then successively leads to the signal energy $E_{D}\left(t,\;d,V_{b}\right)$, the physical action $W_{D}\left(t,\;d,V_{b}\right)$ and finally the physical action $W_{D}\left(\tau_{t},\;d,V_{b}\right)$, received upon termination of the current pulse:

(19) $$W_{D}\left(\tau_{t},\;d,V_{b}\right)=\frac{1}{6}qV_{b}\tau_{t}\left(d,\;V_{b}\right)=\frac{1}{6}\;qV_{b}\;\tau_{min}\;S\left(d,V_{b}\right).$$

In addition to the kinetic energy $E_{kin}=qV_{b}$ that the photoelectron had gained during its transit through the detector gap, the produced physical action also depends on the time $\tau_{t}\left(d,\;V_{b}\right)$ that the electron transit has taken. As, depending on the operational parameters d and $V_{b}$, the transit time can become orders of magnitude larger than the photon vibrational period $\tau_{ph}$, this elongation effect is instrumental in turning microscopic photon detector interactions into macroscopically observable events. In receiving the associated piece of physical action, $W_{D}\left(\tau_{t},\;d,V_{b}\right)$, a price had to be paid. This price, obviously, consisted in the dissipation of the energy that the photoelectron had gained during its transit through the detector gap:

(20) $$S_{D}\left(T_{d},V_{b}\right)=\frac{qV_{b}}{T_{D}}.$$

**Figure 3:**
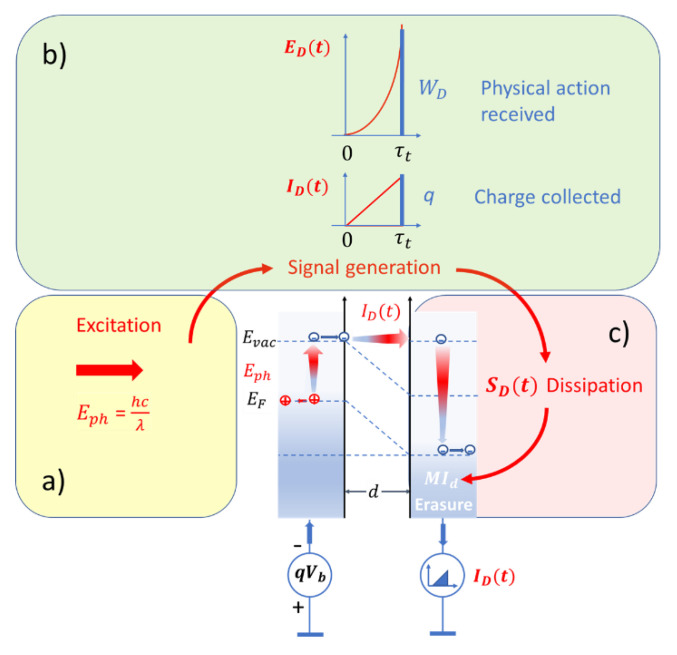
Sequence of processes involved in PID signal generation; (**a**) energy invested; (**b**) value received; (**c**) price paid.

During dissipation the kinetic energy of the photoelectron is broken down into $E_{kin}/k_{B}T_{d}$ pieces of energy of size $k_{B}T_{d}$, which due to the thermal coupling of the PID to the environment, ultimately end up in the environment creating an entropy $S_{D}=qV_{b}/T_{D}$ there. As the environment represents a thermal reservoir of effectively infinite size, this entropy is added to the reservoir’s entropy without producing any measurable increase in temperature. In this way, the produced entropy is turned into a piece of missing information $MI_{D}=S_{D}/k_{B}\ln\left(2\right)$ concerning the internal state of motion within this huge reservoir. Once this has happened, the detection device has been reset to its pre-detection state which readied it for a new round of photon detection. For clarity, this process of turning photon energy into macroscopically observable events, i.e., pieces of physical action at the expense of energy dissipation, is illustrated in Figure 3.

A special situation arises when the bias potential $V_{b}$ is chosen to match the photon energy that had initially been invested to generate the photoelectron, i.e., $qV_{ph}=E_{ph}$. In this case the photon energy is converted in a one-to-one manner into the kinetic energy of a photoelectron and the entropy finally created is the same as if the photon had become directly absorbed in the huge environmental reservoir without producing any macroscopically observable effect at all. Further reducing the detector gap width to its physical minimum, the gain in physical action is:

(21) $$W_{D}\left(\tau_{t},\;d,E_{ph}\right)=\frac{1}{6}h\sqrt{\frac{2m_{e}c^{2}}{E_{ph}}}\gg \hbar ,$$

and its entropic cost:

(22) $$S_{D}\left(T_{d},V_{b}\right)=\frac{E_{ph}}{T_{D}}.$$

**Figure 4:**
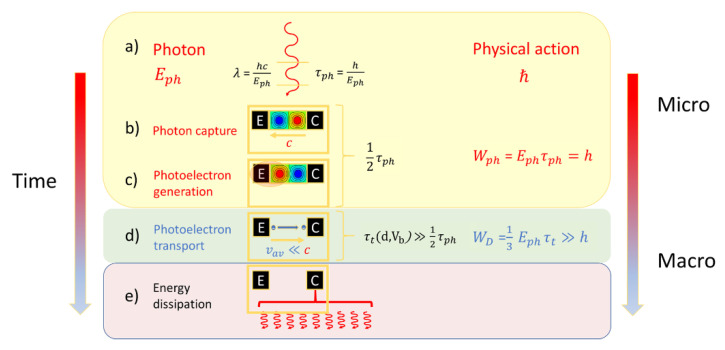
Turning microscopic into macroscopically observable events: Proceeding through the sequence of processes (**a**–**e**), processes become increasingly slowed down and more observable as more physical action is generated.

Realizing that $W_{D}\left(\tau_{t},\;d,E_{ph}\right)\;\gg \hbar$, even in this case, it is revealed that detection involves a fair bit of amplification as the produced physical action considerably overwhelms the physical action that had initially been carried with the photon prior to its detection in the form of its spin angular momentum. As already discussed above, such amplification is necessary to turn a microscopic event—the photon detector interaction—into a macroscopically observable event, i.e., into a current transient. The sketch of Figure 4 tries to visualize this gain process, considering the capture of a photon in a narrow detector gap of size d=λ and its conversion into a photoelectron which moves with a much lower speed than the photon through the gap, thus stretching out the detection event onto a macroscopically observable time scale.

### 4.2. Observational Value of Detection Events

As discussed in Section 2 and Section 3, the observation of a detection event does not uniquely prove that a true photon–detector interaction had taken place. Any observed event therefore is burdened with a limited observational value. In the standard theory this value is measured through the signal-to-noise ratio and other FOMs which build on this central figure of merit [20].

Information as a physically measurable quantity can be introduced into the established theory of detector response simply by using

(23) $$i_{D}\left(E_{ph},\;T_{D},V_{gap},\;V_{b}\right)=\frac{1}{\ln\left(2\right)}\ln\left[SN\left(E_{ph},T_{D},V_{gap},V_{b}\right)\right].$$

Applying this definition to Equation (17), the information gain upon detection $i_{D}$ is seen to consist of three contributions:

(24) $$i_{D}\left(E_{ph},\;T_{D},V_{gap},\;V_{b}\right)=i_{diss}\left(E_{ph},\;T_{D}\right)-i_{loc}\left(E_{ph},\;V_{gap}\right)-i_{time}\left(d,\;V_{b}\right).$$

The first of these,

(25) $$i_{diss}\left(E_{ph},\;T_{D}\right)=\frac{1}{\ln\left(2\right)}\ln\left[\frac{1}{\sqrt{\pi }}\sqrt{\frac{E_{ph}}{k_{B}T_{D}}}\;exp\left[\frac{E_{ph}}{2k_{B}T_{D}}\right]\right],$$

measures the number of energy quanta of size $k_{B}T_{D}$ that can be generated upon dissipation of the photon energy $E_{ph}$ inside a detector operated at a temperature $T_{D}$. In the limit of large photon energies this approximates as

(26) $$i_{diss}\left(E_{ph},\;T_{D}\right)=\frac{1}{2\;\ln\left(2\right)}\frac{E_{ph}}{k_{B}T_{D}}=\frac{1}{2}i_{pot}\left(E_{ph,}T_{D}\right),$$

which corresponds to half the potential information that had initially been carried with the photon itself.

The second contribution adds with a negative sign and measures the information that had been lost due to the incomplete localization of the photon inside the detector gap:

(27) $$i_{loc}\left(E_{ph},\;V_{gap}\right)=\frac{1}{2\ln\left(2\right)}\ln\left[\frac{V_{gap}}{V_{min}}\right].$$

The third term also adds with a negative sign, as after conversion of the photon into a photoelectron, the photoelectron moves with an average speed, vav=12v(τt,d,Vb)≪c, much lower than the speed of light through the detector gap:

(28) $$i_{time}\left(E_{ph},\;d,\;V_{b}\right)=\frac{1}{2\;\ln\left(2\right)}\ln\left[\frac{\tau_{t}\left(d,\;V_{b}\right)}{\tau_{ph}}\right]=\frac{1}{2\;\ln\left(2\right)}\ln\left[\frac{c}{v\left(\tau_{t},d,V_{b}\right)}\right].$$

**Figure 5:**
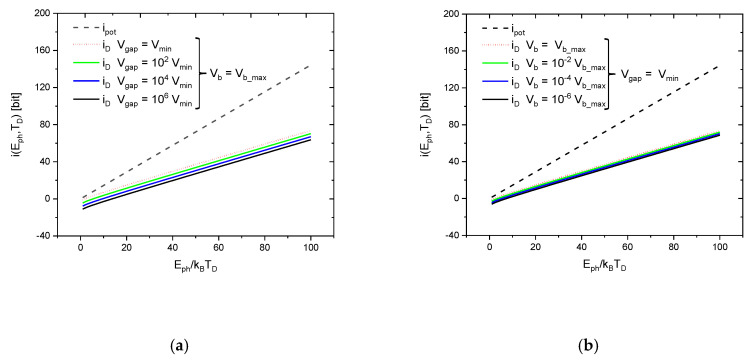
Information gained in detection, $i_{D}$, at different detector operation conditions, compared to the potential information carried by a photon of energy $E_{ph}$ relative to a heat bath with a temperature T equal to the detector operation temperature $T_{D}$. The differently colored curves stand for different detector operation conditions. In (**a**) the effect of gap volume variations is shown while the detector is biased at its maximum possible bias voltage; (**b**) shows the effect of bias potential variations while the detector is operated at its minimum possible gap volume.

Figure 5 displays the variation of $i_{D}\left(E_{ph},\;T_{d},V_{gap},\;V_{b}\right)$ of this technically realizable information gain in quantitative detail as a function of the reduced photon energy $E_{ph}/k_{B}T_{D}$ for different parameter settings of d and $V_{b}$. This first set of results shows that the values of $i_{D}\left(E_{ph},\;T_{d},V_{gap},\;V_{b}\right)$ never exceed the value of $i_{pot}\left(E_{ph},T_{D}\right)$, which in Equation (1) has been proposed to be an intrinsic information content that is carried with the photons themselves before any photon–detector interaction takes place.

**Figure 6:**
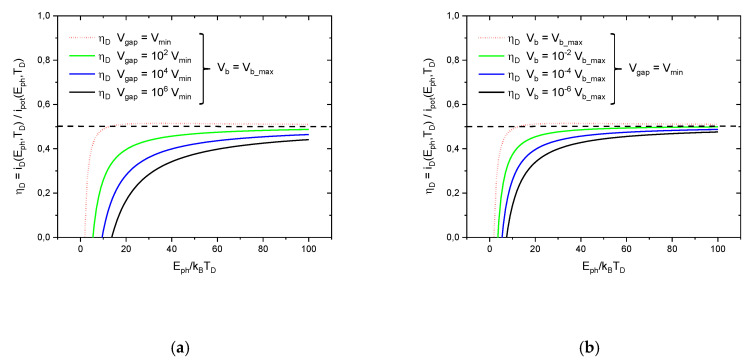
Detection efficiency $\eta_{D}$ as a function of the reduced photon energy. The differently colored curves stand for different detector operation conditions. In (**a**) the effect of gap volume variations is shown while the detector is biased at its maximum possible bias voltage; (**b**) shows the effect of bias potential variations while the detector is operated at its minimum possible gap volume.

Figure 6 displays this same result in a slightly different way, namely as a ratio of realized information gained and information intrinsically carried with the photons themselves.

(29) $$\eta_{D}\left(E_{ph},\;T_{D},V_{gas},\;V_{b}\right)=\frac{i_{D}\left(E_{ph},\;T_{D},V_{gap},\;V_{b}\right)}{i_{pot}\left(E_{ph},\;T_{D}\right)}.$$

This latter FOM more clearly shows that the realized information $i_{real}=i_{D}$ hardly ever exceeds a level of 50% relative to the information $i_{pot}$ that has intrinsically been carried with the photons themselves. The parameter $\eta_{D}\left(E_{ph},\;T_{D},d,\;V_{b}\right)$ measures the detector performance on a simple percentage basis relative to the performance of a hypothetical detector that can fully reveal the potential information carried by the photons themselves. Consequently, the parameter $2\eta_{D}$ $\left(0\leq 2\eta_{D}\leq 1\right)$ may be taken as a measure for the observational value that is associated with a detection event that had been generated under the parameter settings listed in the function $\eta_{D}\left(E_{ph},\;T_{D},V_{gap},\;V_{b}\right)$.

In the Appendix A it is shown that the FOM of detection efficiency (Equation (29)) maps onto the more commonly used FOM of specific detectivity $D_{ideal}^{*}$ of ideal photon detectors, as described in the textbook of R.H. Kingston [20]. As this detectivity represents an upper limit for the values of $D^{*}$ that are technologically achievable with other kinds of photon detectors, the results of Figure 5 and Figure 6 are of a more general value, extending beyond the PIDs considered above. These results, therefore, support the point of view that the information $i_{pot}\left(E_{ph},\;T_{D}\right)$ does indeed represent an intrinsic level of information that is carried with photons of energy $E_{ph}$ themselves prior to undergoing a detection process in a detector operated at a temperature $T_{D}$. Specifically, Figure 6 reveals that, in agreement with Equation (2), this intrinsic information can only partially be recovered as realized information in a technical device:

(30) $$i_{real}\left(E_{ph},\;T_{D}\right)=i_{D}\left(E_{ph},\;T_{D}\right)\leq i_{pot}\left(E_{ph},\;T_{D}\right)=\frac{1}{\ln\left(2\right)}\frac{E_{ph}}{k_{B}T_{D}}.$$

As both realized and potential information are entropies which result from the dissipation of photon energy, this latter equation proves that the process of information gain is inherently irreversible.

Equation (30), in particular, shows that gaining a single bit of realized information minimally requires an input in photon energy of

(31) $$E_{min}\geq k_{B}T_{D}\ln\left(2\right),$$

units of energy. Such an energy cost of information has originally been proposed by Brillouin [8] to represent the minimum energy cost that needs to be paid for gaining one bit of information. Landauer, later argued that this same amount of energy needs to be expended for erasing one bit of stored information [9]. As detection events, i.e., pieces of realized information, are of a transient nature with lifetimes corresponding to the detector response time, such transients are somewhat self-erasing. Consequently, the energy invested in the generation of the information $i_{real}\left(E_{ph},\;T_{D}\right)$ is immediately turned into missing information as the photon energy is dissipated inside the detector and thereby ultimately lost. It therefore appears that in photon detection, the Brillouin and Landauer limits for information gain and information erasure map onto each other, producing the same predictions for the per-bit energy costs of information gain and information erasure.

## 5. Summary and Conclusions

In this section we want to summarize and generalize beyond the special kind of PID detectors considered above. 

The central result of our paper is contained in Equation (30), which shows that the information $i_{pot}\left(E_{ph},T_{D}\right)$ represents an upper limit of information gain that cannot not be revealed and turned into realized information, $i_{real}\left(E_{ph},T_{D}\right)$, not even by ideal photon detectors. Equation (30), therefore supports the point of view, raised in the introduction, that $i_{pot}\left(E_{ph},T_{D}\right)$ does indeed represent a piece of information that is intrinsically carried with photons of energy $E_{ph}$ themselves and as valued relative to a potential detector operated at a temperature $T_{D}$.

Potential information has been shown to physically relate to the ability of a photon of energy $E_{ph}$ of generating entropy $S_{D}=E_{ph}/T_{D}$ as it interacts with a macroscopic piece of matter maintained at a finite temperature $T_{D}$. When this matter takes the form of a macroscopic heat reservoir with internal energy $U\gg E_{ph}$, the produced entropy is added to the reservoir´s entropy without any macroscopically measurable change in the reservoir temperature $T_{D}$. In such an interaction, the photon´s potential information is completely converted into missing information $\Delta MI_{D}=\Delta S_{D}/k_{B}\ln\left(2\right)$ about the internal state of motion inside the reservoir and is thereby ultimately lost.

A different situation arises when the photon becomes absorbed inside a photon detector. Such devices are constructed in a way that macroscopically observable events are produced as the photon energy becomes internally dissipated. Such events, therefore, form macroscopic observational images of the microscopic photon–detector interactions inside such devices. In technologically important devices, such events take the form of electrical signal transients. As, however, output signals need not necessarily involve electrical energy, we have proposed in Section 4.1 that physical action (Wirkung), i.e., the result of work done, may represent a more generally valid physical equivalence of the conceptional vehicle of a “detection event”.

As macroscopically observable events can also arise from internally generated blackbody radiation, each and every observed event has a finite observational value. We have shown in Section 4.2 that this observational value can be expressed by applying the Shannon definition of entropy [6] to the conventionally derived signal-to-noise ratio. In this way a value of statistical significance, $i_{real}\left(E_{ph},T_{D}\right)$, is obtained that measures the probability that an observed event is due to a true photon–detector interaction and unlikely due to a random thermal fluctuation inside the detector itself.

**Figure 7:**
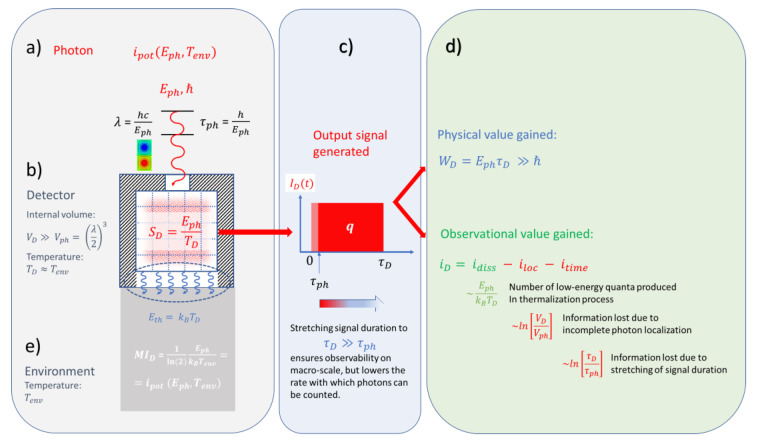
Photon detectors acting as thermodynamic engines converting potential into realized information: (**a**) photon to be detected, (**b**) photon interacting with detector filled with a background of internally generated thermal radiation; (**c**) output signal transient generated; (**d**) informational value gained; (**e**) energetic and entropic cost paid.

As both realized and potential information are physically measurable entropies, Equation (30) shows that information gain in photon detection represents a thermodynamically irreversible process. Overall, this equation suggests that photon detectors may be regarded as thermodynamic engines that convert, with limited efficiency, potential information into pieces of realized information. This idea of an information-generating thermal device is pictorially illustrated in Figure 7.

## Appendix A. Technical Figures of Merit for Photon Detectors

So far, our arguments have been based on considerations focusing on a specific form of photon detector, i.e., a photo-ionization detector (PID). In order to show the more general applicability of our results, we calculate the specific detectivity $D_{PID}^{*}$ of our PID and show that this conventional FOM directly maps on the detectivity $D_{ideal}^{*}$ of an ideal photon detector as described in the textbook of R.H. Kingston [20]. As $D_{ideal}^{*}$ represents an upper limit to the performance of all kinds of technical photon detectors, evidence is presented that $i_{pot}\left(E_{ph},T_{d}\right)$ is the absolute maximum of $i_{D}\left(E_{ph},T_{D}\right)$ that can be realized with any kind of technical device.

Before turning to this calculation, we note that the detection efficiency $\eta_{D}$ relates to the specific case of single-photon detection. The conventional figures of merit, in contrast, relate to cases in which the sensor signal derives from the absorption of a large number of photons. FOMs in common usage are:

noise-equivalent power:

(A1) NEP,

detectivity:

(A2) $$D=\frac{1}{NEP},$$

specific detectivity

(A3) $$D^{*}=\frac{\sqrt{A}\sqrt{B}}{NEP}.$$

In Equation (A1) the noise-equivalent power, NEP, is defined as the photon input power that produces a signal-to-noise ratio of SN=1 in the specific device. Equation (A2) is a simple measure of detector sensitivity based on the noise-equivalent power. Both NEP and detectivity, D, depend on the specific kind of transducer principle and on parameters relating to the specific detector design (e.g., the detector area A) and detector operation parameters (detector operation temperature $T_{D}$ and electronic filter band width B), chosen in the specific application scenario. The specific detectivity, defined in Equation (A3), finally, removes the dependence on these design and operation-specific parameters and focuses more directly on the transducer principle as such.

With these considerations in mind, we now turn to the evaluation of the NEP of our PID. In order to attain a signal-to-noise ratio equal to one, the root-mean-square (rms) number of noise electrons, $N_{n}$, needs to match the number of signal electrons, $N_{s}$:

(A4) $$N_{s}\left(T_{D}\right)=\sqrt{N_{n}\left(T_{D}\right)}=\sqrt{A}\sqrt{\tau_{t}\left(d,V_{b}\right)}\sqrt{n_{th}\left(E_{ph},T_{D}\right)}$$

As in the main text above, A stands for the area of the emitter and collector electrodes and $\tau_{t}\left(d,V_{b}\right)$ for the electron transit time through the detector gap.

The number of signal electrons, on the other hand, is related to the number of signal photons $N_{ph\_s}$ by

(A5) $$N_{s}=\eta_{PID}\;N_{ph\_s}$$

with $\eta_{PID}$ standing for the quantum-efficiency with which signal photons become converted into signal electrons. As each signal photon contributes a fraction of $E_{ph}/\tau_{t}\left(d,V_{b}\right)$ to the total input photon power, the noise-equivalent power becomes:

(A6) $$NEP_{PID}\left(A,\;d,T_{D},V_{b}\right)=\frac{1}{\eta_{PID}}\left(E_{ph}/\tau_{t}\left(d,V_{b}\right)\right)N_{s}(T_{D}).$$

This minimum detectable photon power still depends on the detector-specific design parameters A and d and the sensor operation parameters $T_{D}$ and $V_{b}$. Turning to the calculation of $D_{PID}^{*}$, a FOM is obtained, which solely depends on the sensor operation temperature $T_{D}$ and the photon energy $E_{ph}$:

(A7) $$D_{PID}^{*}\left(T_{D}\right)=\eta_{PID}\frac{\sqrt{A}\sqrt{\frac{1}{\tau_{t}\left(d,V_{b}\right)}}}{\frac{E_{ph}}{\;\tau_{t}\left(d,V_{b}\right)}\;\sqrt{A}\sqrt{\tau_{t}\left(d,V_{b}\right)}\sqrt{n_{th}\left(E_{ph},T_{D}\right)}}=\eta_{PID}\frac{1}{E_{ph}\;\sqrt{n_{th}\left(E_{ph},T_{D}\right)}\;}$$

With $n_{th}\left(E_{ph},T_{D}\right)$ being given by Equation (7), one finally obtains:

(A8) $$D_{PID}^{*}=\eta_{PID\;}\sqrt{\frac{c^{2}h^{3}}{2\;\pi }}\frac{exp\left[\frac{E_{ph}}{2\;k_{B}T_{D}}\right]}{\;{\left(k_{B}T_{D}\right)}^{5/2}\frac{E_{ph}}{k_{B}T_{D}}\sqrt{\left\{{\frac{E_{ph}}{{\left(k_{B}T_{D}\right)}^{2}}}^{2}+2\frac{\;E_{ph}}{k_{B}T_{D}}+2\right\}}\;}=\eta_{PID}\;D_{ideal}^{*},$$

i.e., a result that exactly matches the detectivity $D_{ideal}^{*}$ of an ideal photon detector [20], except for the lowered quantum efficiency of $\eta_{PID\;}=1/2$, which arises from the fact that, on average, only every second signal photon becomes absorbed inside the emitter electrode from where signal photoelectrons can be emitted. In the informational reformulation (Equation (30)) this difference in quantum efficiency shines up in the form that $i_{real}\approx 0.5\;i_{pot}$.
